# Integrated single- and two-photon light sheet microscopy using accelerating beams

**DOI:** 10.1038/s41598-017-01543-4

**Published:** 2017-05-03

**Authors:** Peeter Piksarv, Dominik Marti, Tuan Le, Angelika Unterhuber, Lindsey H. Forbes, Melissa R. Andrews, Andreas Stingl, Wolfgang Drexler, Peter E. Andersen, Kishan Dholakia

**Affiliations:** 1 SUPA, School of Physics and Astronomy, University of St Andrews, North Haugh, St Andrews KY16 9SS UK; 2 Institute of Physics, University of Tartu, W. Ostwald St 1, Tartu, 50411 Estonia; 3 Department of Photonics Engineering, Technical University of Denmark, Frederiksborgvej 399, 4000 Roskilde, Denmark; 4 Femtolasers Produktions GmbH, Fernkorngasse 10, 1100 Vienna, Austria; 5 Center for Medical Physics and Biomedical Engineering, Medical University Vienna, Waehringer Guertel 18-20, 1090 Vienna, Austria; 6 School of Medicine, University of St Andrews, North Haugh, St Andrews KY16 9TF UK

## Abstract

We demonstrate the first light sheet microscope using propagation invariant, accelerating Airy beams that operates both in single- and two-photon modes. The use of the Airy beam permits us to develop an ultra compact, high resolution light sheet system without beam scanning. In two-photon mode, an increase in the field of view over the use of a standard Gaussian beam by a factor of six is demonstrated. This implementation for light sheet microscopy opens up new possibilities across a wide range of biomedical applications, especially for the study of neuronal processes.

## Introduction

Light sheet fluorescence microscopy (LSFM), often termed selective plane illumination microscopy (SPIM)^1^ is finding key applications in neuroscience and developmental biology. This imaging modality offers significantly reduced photobleaching in tandem with fast data acquisition using wide field camera technology and is poised to displace conventional confocal and two-photon microscopy.

Light sheet imaging may operate in both single- and two-photon modes. Single-photon light sheet imaging can easily be implemented due to the broad availability of affordable light-sources. However, the excitation light, typically in the blue and green region of the spectrum, undergoes significant scattering when used in biological tissue. Two-photon light sheet imaging offers enhanced penetration depth due to the longer and therefore less scattered wavelength used for excitation. Notably, single-photon light sheet microscopy used for studies of neuronal processes, e.g., in zebrafish, requires an extended illumination at a wavelength (488 nm) that lies within the most sensitive region of the fish’s visible spectrum. This in turn may lead to direct stimulation of the blue photoreceptors in the fish retina, amongst other photosensitive cells, if not taken into consideration by careful design of the imaging geometry^2^. Thus, when studying live zebrafish, two-photon light sheet would have additional and significant advantages^3^.

Advanced geometries for light sheet microscopy include the use of multiple illumination beams^4^ (to obviate shadowing effects) and digital scanning^5^. In particular, two-photon light sheet excitation can be used both in stationary and scanned modes, where the light sheet is created either using a cylindrical lens^6^ or by rapidly scanning a focused beam^7^. To overcome the trade-off between the field of view (FOV) and the axial resolution of the light sheet microscope usually associated with Gaussian beams in the two-photon excitation mode, double-sided illumination^7, 8^ and scanning the focus of tightly focused beam^9, 10^ have both been used. Recently a two-photon Gaussian light sheet microscope in iSPIM configuration has been demonstrated for imaging thick biological samples^11^.

Further innovations that have gained major attention for light sheet imaging include the use of propagation invariant beams. Most notably, Bessel and Airy modes are powerful for achieving wide FOV with sustained high resolution, yet these remain the remit of highly specialised, complex systems^12–16^. Bessel beams are symmetrical and require confocal line scanning in the detection, or structuring (creating period arrays) in light sheet excitation for single photon imaging. Utilizing objectives with NA much higher than usually used in light sheet microscopy (NA 1.1 for detection and NA 0.65 for excitation), structured two-photon Bessel beam light sheet has shown to reach 0.45 μm axial resolution over 50 μm FOV^17^. Benefiting from near-infrared excitation wavelengths and its “non-diffracting” nature, two-photon Bessel beam light sheet can penetrate up to 500 μm into zebrafish embryo delivering 0.5 μm lateral and 2 μm axial resolution over 600 μm wide FOV^18^.

A step change for this field would be the realisation of a light sheet microscope as a facile, compact system, that would obviate any scanning and in addition readily interchange between single- and two-photon imaging, whilst at the same time exploiting the benefits of propagation invariant fields. In this letter, we achieve this goal with an integrated single- and two-photon microscope in a new geometry utilising accelerating Airy beams. We use newly developed fs laser systems as part of our system to realise a small footprint for our microscope.

It is interesting to comment on the choice of Airy beams versus Bessel beams for this goal. Energy efficient generation of Bessel beams with high quality requires an optical set-up that involves an axicon, a focusing lens and annuli^13^. Furthermore, the Bessel beam light sheet is intrinsically a digitally scanned one, requiring a scanning element for creating a virtual light sheet. This can be impractical in certain situations. In contrast, Airy beams can readily be generated in both 1D and 2D^19^, and have successfully been used for a single-photon fluorescence light sheet imaging^16, 20^. It is very important to stress that the side lobes for the Airy beam in fact contribute to the final image formation, in contrast to the Bessel beam where this is not the case for single photon mode. The Airy light sheet can be generated using a spatial light modulator, a custom fabricated cubic phase mask or very straightforwardly using a tilted cylindrical lens, and a focusing lens or objective^20^. In two-photon imaging one must also take into account the dispersion and the polychromatic nature of the ultrashort pulses. Depending on the generation method, ultrashort pulsed Airy beams have different propagation characteristics^21, 22^. In the current application it is desirable to use a cubic phase mask located in a conjugate pupil plane, so that all the wavelength constituents of the pulse will travel along the same curved trajectory. The benefit of non-scanning Airy mode light sheet implementation is that it is more facile to implement and therefore brings for the first time propagation invariant fields to wider use in light sheet imaging.

**Figure 1:**
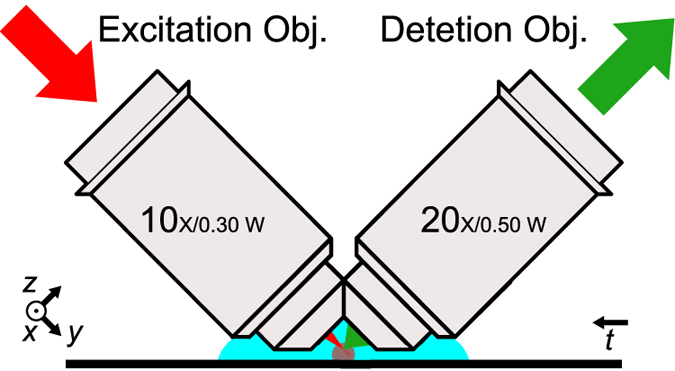
The iSPIM geometry of light sheet microscopy used in this paper allows to image tissue samples mounted on standard microscope slides. The *xyz* coordinate system used throughout this paper is defined as relative to the detection objective, the *t*-axis represents translation axis.

Light sheet microscopes can be built in many different configurations, including multiview and multiexcitation configurations^23^. The traditional SPIM-arrangement^1^, where the sample is suspended from above, can be awkward for most microscopy samples mounted on standard microscopy slides. For targeting applications in neuroscience, we have chosen the 45° iSPIM configuration^24^ which accepts tissue samples mounted on standard microscope slides as shown in Fig. 1.

## Results

**Figure 2:**
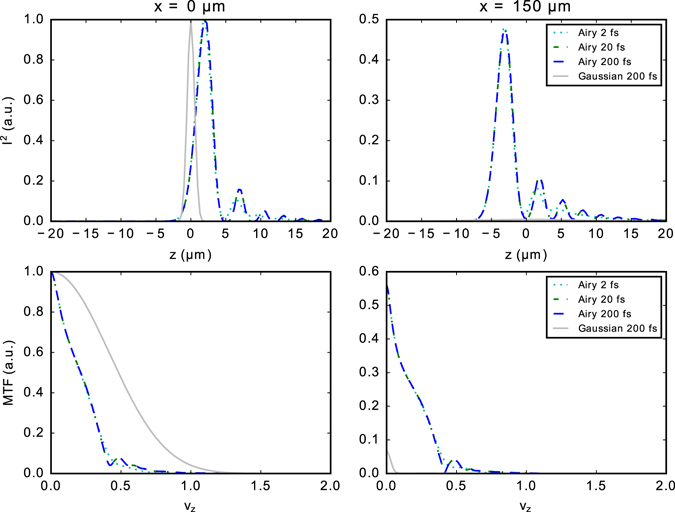
Simulated excitation intensity squared point spread functions (I^2^) and corresponding modulation transfer functions (MTF) for the two-photon Gaussian and Airy light sheets (*α* = 7) with different pulse durations and a Gaussian spectrum. The plots are shown at the waist *x* = 0 μm and at distance *x* = 150 μm from the waist. The spatial frequency *ν*_*z*_ is normalized to maximum transmitted spatial frequency 2*NA*/*λ*, numerical aperture *NA* = 0.3 and excitation wavelength *λ* = 800 nm.

First, we simulated the excitation point spread functions (PSF) and corresponding modulation transfer functions (MTF) for the two-photon Gaussian and Airy light sheets with different pulse durations and a Gaussian spectrum (Fig. 2). It can be seen that the PSF for 20 fs and 200 fs pulsed Airy pulses is nearly identical and the axial resolution characteristics does not depend on the pulse length unless the pulse duration is below ~5 fs.

**Figure 3:**
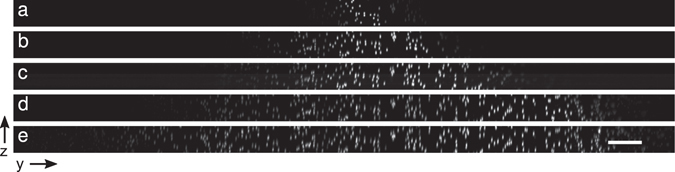
Maximum intensity projection along *x* axis of a centre region of a scan of 0.4 μm diameter green fluorescing polystyrene microspheres embedded in agarose with different illumination parameters: (**a**) Gaussian light sheet, (**b**–**e**) Airy light sheet with increasing *α* parameter (3.5, 7, 14, and 21) for cubic phase modulation depth. Imaged in two-photon mode using the Core Laser oscillator, illumination from left. Scale bar 20 μm.

We then tested the performance of two-photon fluorescence light sheet microscope under different illumination types using small, 0.2 μm and 0.4 μm fluorescent microspheres. Comparison between Gaussian and Airy beams with the same numerical aperture (NA 0.3) of the excitation beam shows that the use of pulsed Airy beam illumination for two-photon fluorescence excitation allows to extend the light sheet at least six fold in the propagation direction to approximately 300 μm, compared to a FOV of 30 μm that is achieved for Gaussian light sheet in the current configuration of the system, as is shown in Fig. 3. Corresponding light sheet profiles for different illumination parameters are shown in Supplementary Fig. S2.

The width of the light sheet is determined by the focal length of the cylindrical lens used to create the light sheet. For *f* = 50 mm this corresponds to a width of approximately 300 μm in the current configuration. This is independent of the light sheet type used for the illumination.

The lateral resolution of the light sheet microscope is determined by the detection objective and tube lens combination. The effective lateral sampling rate in our system was 325 nm, while the Nyquist sampling rate for NA 0.5 widefield microscope would be 250 nm. Experimentally the lateral resolution of the system was measured over the ~300 × 300 μm^2^ FOV to be 0.91 ± 0.01 μm (FWHM). This is calculated as a mean over all the tested beam types, which includes Gaussian light sheet and Airy light sheets with different cubic modulation parameters which were probed with green fluorescing beads.

The obtainable axial resolution is dependent on the light sheet type and the laser wavelength used. The increase of the FOV comes at the expense of the maximum achievable axial resolution. For the Gaussian light sheet, a peak axial resolution of 1.4 μm was achieved for the 780 nm Core laser and 1.9 μm for the 1045 nm HighQ-2 laser as determined experimentally measuring the PSF on fluorescent microspheres. For the Airy light sheets, the axial resolution is dependent on the cubic modulation parameter *α* which on the other hand determines also the FOV. For obtaining the widest FOV (~300 × 300 μm), the axial resolution is decreased to 2.6 μm (3.0 μm for the HighQ-2). For a Gaussian light sheet with the same FOV the axial resolution would be decreased to ~4 μm. The experimentally determined axial resolution measurement results are summarized in Table 1. Using a cubic phase mask with modulation depth of *α* = 7 (axial resolution 1.9 μm) or *α* = 14 (axial resolution 2.2 μm) with corresponding FOV of 153 μm or 222 μm provides good compromise between increased FOV and axial resolution in the current application.

**Table 1:** Comparison of the experimentally determined axial resolution and FOV for each light sheet type in microns.

Excitation Laser	Gaussian	Airy (*α* = 3.5)	Airy (*α* = 7)	Airy (*α* = 14)	Airy (*α* = 21)
Core (*λ* = 780 nm)	1.42 ± 0.03	1.86 ± 0.11	1.92 ± 0.03	2.22 ± 0.04	2.57 ± 0.11
HighQ-2 (*λ* = 1045 nm)	1.94 ± 0.02	2.11 ± 0.06	2.43 ± 0.09	2.64 ± 0.13	2.96 ± 0.19
FOV	47 ± 12	97 ± 17	153 ± 17	222 ± 42	288 ± 71

The axial resolution is calculated as a mean FWHM of PSF as probed with 0.2 μm diameter green and red fluorescent microspheres. The FOV is averaged over results obtained with both the Core and HighQ-2 laser. The uncertainties are given at 95% confidence level.

**Figure 4:**
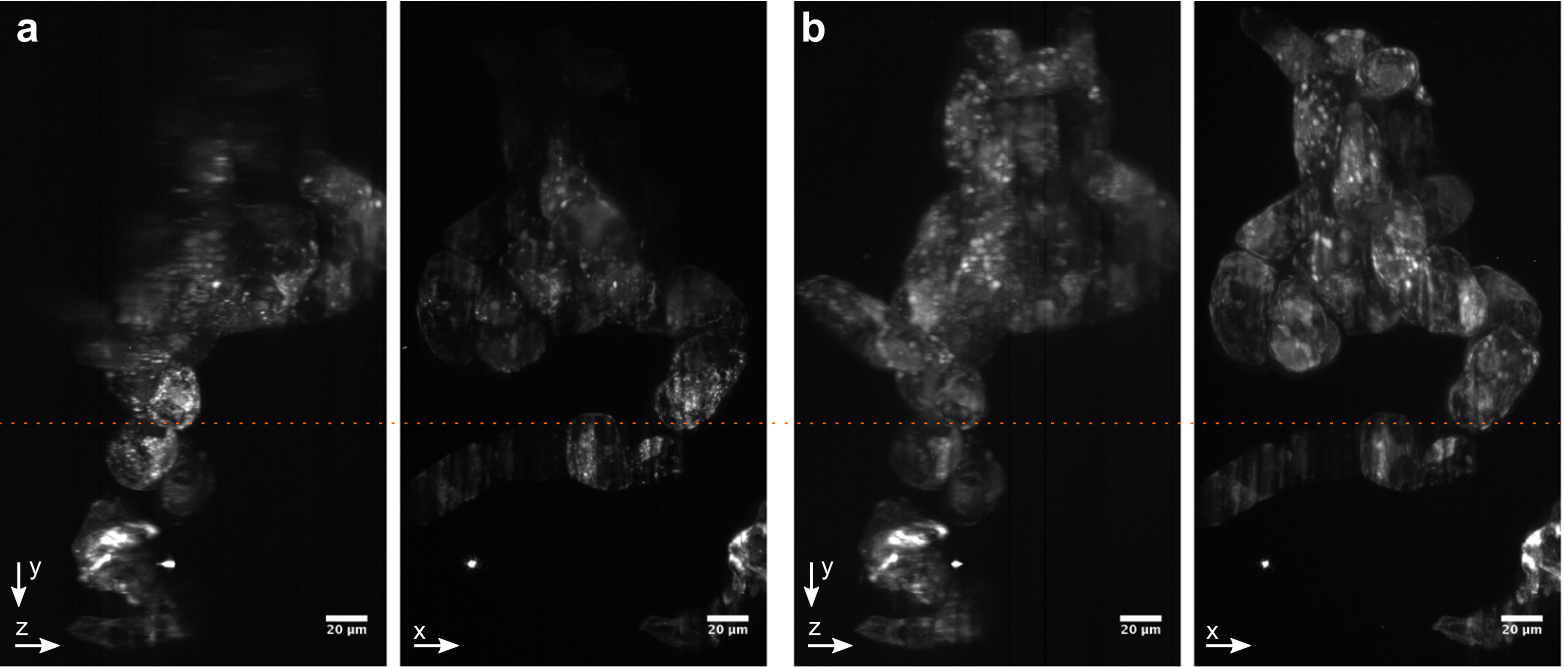
Maximum intensity projections along *x* and *z* axes of an agarose embedded cluster of BY-2 cells expressing actin-RFP. (**a**) Gaussian light sheet, (**b**) Airy light sheet (*α* = 7). Imaged in two photon mode using the HighQ-2 laser, scale bar 20 μm. The dashed orange line shows the approximate position fo the focal plane of the excitation objective.

Furthermore, the microscope system was tested with various biological specimens. Figure 4 shows an example image of a cluster of tobacco Bright Yellow 2 (BY-2) cells. The cell line was obtained form The James Hutton Institute and had been genetically modified to express actin-RFP. Comparing the experimental results from two-photon Gaussian and Airy light sheets it can be seen that although the Gaussian light sheet can extract greater detail in the focal region, the use of Airy light sheet allows us to achieve decent resolution over a much larger FOV.

**Figure 5:**
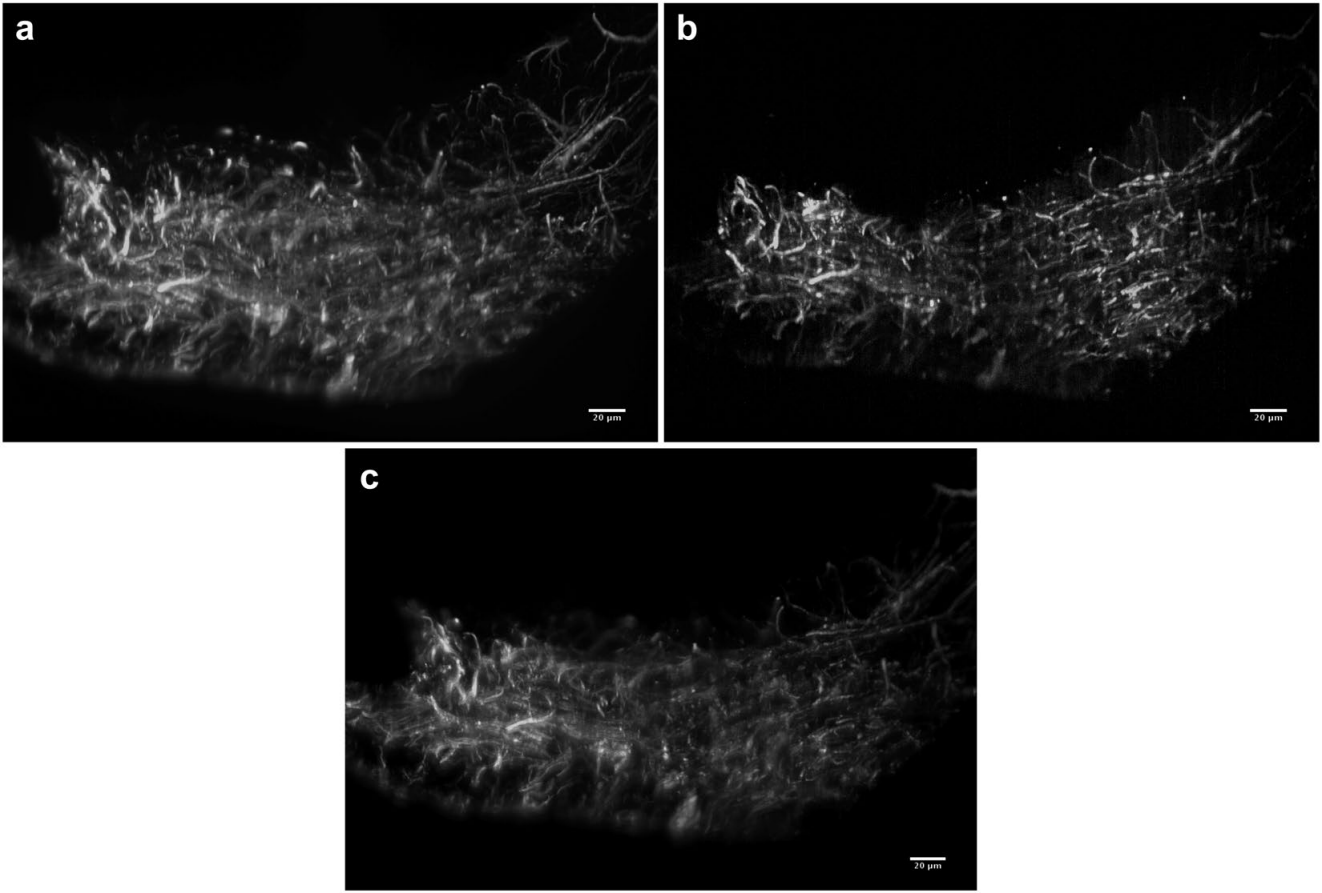
Maximum intensity projections of light sheet image stacks of a thin sagittal slice of fixed rat brain with Alexa Fluor 568 NCAM-immunolabelled axons imaged in two photon mode using the HighQ-2 laser. The brain slice was mounted on uneven surface. (**a**) Gaussian light sheet, (**b**) Airy light sheet (*α* = 7). (**c**) The same region imaged in single-photon Airy light sheet mode (excitation wavelength 488 nm, *α* = 7, deconvolved). Scale bars 20 μm, gamma adjusted for visualization. See Supplementary Videos 1–3.

A particular interest in developing two-photon light sheet system lies in its possible applications in neurological imaging. Therefore, the system was tested with fixed rat brain slices as shown in Fig. 5. It can be seen that the two-photon light sheet can easily penetrate through the entire 40 μm thick sample in both the Gaussian (5a) and the Airy (5b) configuration. In that particular projection it can be seen the Gaussian light sheet provides better resolution in the top right corner of the image compared to the Airy light sheet, however the Airy light sheet achieves better overall resolution over the entire FOV. The corresponding image of the same region of the sample obtained using single-photon Airy light sheet is shown in Fig. 5c.

**Figure 6:**
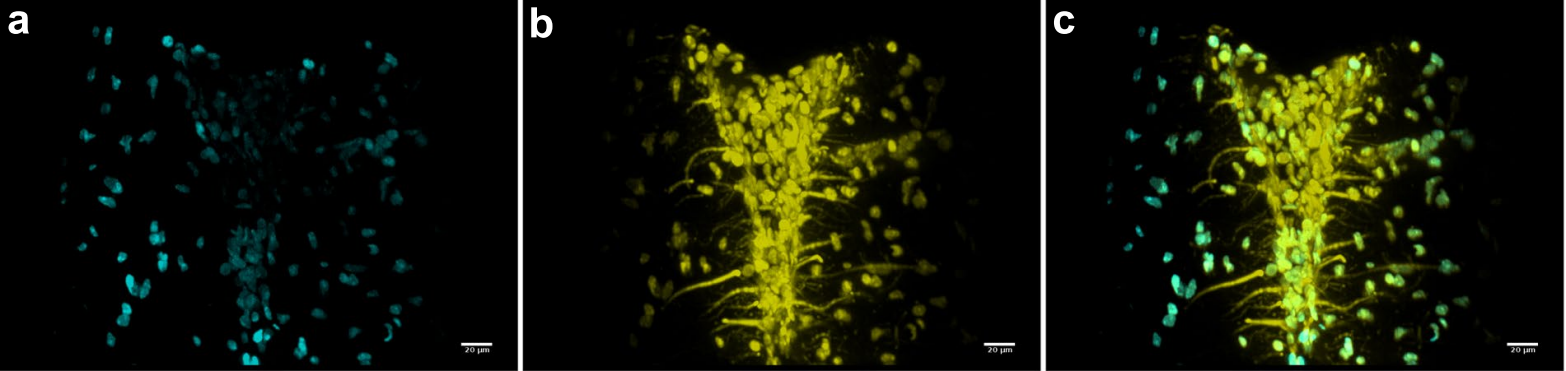
Maximum intensity projections of light sheet image stacks of a thin sagittal slice of fixed rat brain with Alexa Fluor 568 NCAM-immunolabelled axons imaged in two photon mode using M-Squared Sprite-XT laser. (**a**) DAPI channel, (**b**) Alexa Fluor 568 channel, (**c**) Composite image. Scale bars 20 μm. See Supplementary Videos 4 and 5.

Figure 6 demonstrates a simple two-colour imaging mode for the system using the M-Squared Sprite-XT ultrafast oscillator. The images of a brain slice for different fluorescent channels were obtained by subsequent volume scans, changing the emission filter. In this particular case, the laser wavelength was the same for both channels and set to 770 nm, but this could in principle be optimized for each fluorophore.

## Discussion

From our simulations, one can see that the PSF only weakly depends on the pulse duration. The advantage of using shorter pulses would be that these can reach higher peak powers at the same average laser power, with the price of requiring better dispersion compensation. This finding relaxes the requirements of the fs-oscillator.

Using an Airy light sheet for excitation, the FOV can be drastically enlarged to about 300 × 300 μm^2^, while only slightly compromising the axial resolution to about 3 μm. Furthermore, the FOV and the axial resolution can interdependently be tuned to the experimentally required size, by using a phase mask with a different *α* parameter. The system presented here thus proves advantageous in comparison to what is currently found in the literature, both in FOV and axial resolution as well as, importantly, in the fact that no scanning is needed. Calculating a dimensionless figure of merit, (depth of field × width of lightsheet)/(axial resolution^2^), for comparison of lightsheet microscopy systems, our system yields a value of about 10,000, while non-scanned systems employing Gaussian beams yield about 500^6, 11^. Adding the complexity of a digitally scanned lightsheet system, these low values can be increased to 1,000–5,000 employing Gaussian beams^3, 8, 25^, and to 10,000–50,000 employing Bessel beams^15, 18^. The advantage of Bessel beams over Gaussian beams in scanned systems has already been pointed out by Mertz^26^.

For the Airy light sheet used in single-photon excitation it is necessary to have 5–6 Airy side lobes within the depth of focus of the detection objective for the best axial resolution through deconvolution^16^. However, in the two-photon regime this is not required, as the side lobes are much weaker for the two-photon Airy beam. The best axial resolution for a given Airy beam can be found by optimally filling the back aperture of the excitation objective and matching the depth of focus of the detection objective with the width of the main lobe of the Airy light sheet. Therefore, the two-photon Airy light sheet does not exhibit the same effective use of the overall photon budget as in the single-photon case as only the main lobe of the two-photon Airy light sheet is used effectively in the image formation. The benefit arises here from the longer excitation wavelengths and having a better axial resolution over larger FOV compared to Gaussian illumination.

We have shown this by imaging both tobacco cells and rat brain slices. The Airy light sheet also provides an important advantage when imaging thin slices, as show in Fig. 5a and b: while the Gaussian light sheet can provide the best resolution over a very limited FOV, the wider FOV of the Airy light sheet easily alleviates imaging errors when the sample cannot be kept exactly in the focus of the illumination objective during a *z*-stack or when the sample is placed on uneven surface. As it is easy to switch between Gaussian and Airy light sheets in the setup, the system is reconfigurable for the particular imaging requirements. Additionally, it is possible to combine two-photon excitation with single-photon excitation using the same setup.

We have utilized recently developed ultra-compact fs-sources, making the system portable. Additionally, these lasers do not require the presence of a specialist laser engineer, enabling deployment to a wider user base. However, the fixed-wavelength sources (780 nm and 1045 nm) limit the addressable fluorophores (mostly blue/cyan and yellow/red dyes respectively). To access a wider range of fluorophores, the system can readily be expanded by coupling other fs-sources suitably into the beam path, with the cost of an increased footprint of the system. Using a cubic phase plate to generate the Airy light sheet is advantageous in this context, as the light sheets for different wavelengths will be generated along the same trajectory.

In conclusion, we have presented a compact, powerful and flexible platform for simultaneous single photon and two-photon light sheet microscopy, and have shown its abilities in both biological and neurological imaging tasks.

## Methods

### Light sheet microscopy setup

**Figure 7:**
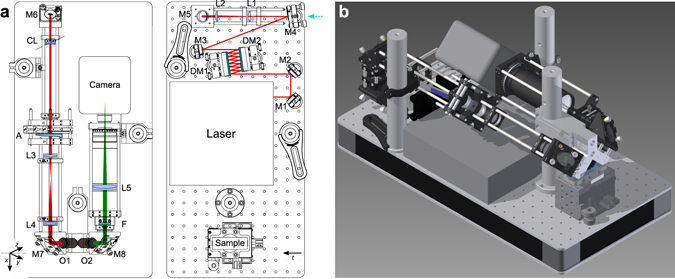
Optical setup with the Femtolasers Core laser oscillator. (**a**) Excitation and detection beam paths and the placement of the optical elements on 30 × 60 cm^2^ breadboard in scale for using the Femtolasers Core laser oscillator. HighQ-2, M-Squared Sprite and CW lasers were coupled into the system by flipping mirror M4 (as shown by the cyan arrow). The *xyz* coordinate system is indicated as relative to the detection objective, the *t*-axis represents translation axis. The axis of the cylindrical lens CL is indicated by the dashed line. (**b**) Computer rendering of the setup. M1–M8 mirrors; DM1, DM2 dispersive mirrors; L1–L5 lenses; CL cylindrical lens; O1 excitation objective; O2 detection objective; F filters; details are given in the Methods and in the Supplementary Table S2.

The developed multiphoton fluorescence light sheet microscope is shown in Fig. 7. The compact optical setup of the microscope was designed to fit onto a 30 × 60 cm^2^ optical breadboard that includes the diode-pumped Core femtosecond oscillator developed by Femtolasers (now Spectra-Physics) or a commercially available Spectra-Physics HighQ-2 oscillator. The Core laser provides 20 fs pulses with a central wavelength of 780 nm and 190 mW average power at 300 MHz repetition rate, while the HighQ-2 laser provides 250 fs pulses at 1045 nm with an average power of 1.5 W at 63 MHz repetition rate. Both lasers have dimensions of <23 × 23 cm^2^ and fit integrally onto the same breadboard with the light sheet microscope. The 780 nm Core laser allows targeting mainly BFP and CFP, whereas the 1045 nm HighQ-2 laser is suitable for RFP and YFP. In addition, the microscope system has been tested with an M-Squared Lasers Sprite-XT tunable fs-oscillator, for accessing a wide range of multi-photon excitation fluorophores in the range of 720–960 nm with a maximum power of 1.8 W at 80 MHz repetition rate. For single-photon excitation, a Coherent Sapphire laser (*λ* = 488 nm, maximum power 20 mW) is coupled into the system using a flip mirror.

The light sheet fluorescence microscope is designed in the 45° iSPIM configuration to accept samples mounted on standard microscope slides^24^. The optical setup is laid out on two levels: the first one that lies on the breadboard includes the laser, a pulse pre-compressor using two dispersion compensating chirped mirror (DM1 and DM2, 7 reflections off each mirror, GDD −250 fs^2^ per reflection), and a Galilean 2× beam expander (L1, *f* = −75 mm UVFS plano-concave lens, L2, *f* = 150 mm UVFS plano-convex lens). The second level is supported by three 1.5 inch posts and is constructed with standard cage-system components that attaches to a custom designed objectives mount. In this configuration, a static light sheet is formed by a cylindrical lens (CL, *f* = 200 mm UVFS plano-convex). Lenses with a focal length between 50–200 mm can be used to provide the optimum width of the light sheet. With the cylindrical lens the light is focused to a line on the Airy-generating cubic phase mask (A). The phase mask is imaged with a 2× expanding telescope (L3, *f* = 50 mm achromatic lens and L4, *f* = 100 mm achromatic lens) onto the back aperture of the excitation objective (O1, Olympus 10×, NA 0.3, water dipping). Protected silver mirrors are used on the excitation beam path M1–M7. M8 is a dielectric mirror for visible wavelengths. M7 and M8 are elliptical in shape to allow to use the full NA of the objectives. The detection pathway consists of the detection objective (O2, Olympus 20×, NA 0.5, water dipping) and an achromatic tube lens (L5, *f* = 180 mm). Both a Thorlabs short-pass filter FESH0700 and a Semrock short pass filter FF01-650/SP-25 have been used in the filter holder F to block the infrared excitation light. For measurements with the Core laser an additional fluorescence filter (Semrock FITC-BP01-Clin-25) was used. Optimal dispersion compensation was found by adjusting the number of reflections off the dispersive mirrors in order to maximize the two-photon fluorescence signal level in fluorescein. The exact imaging conditions for the figures are given in the Supplementary Table S2.

Samples were mounted on microscope slides that were kept in place with a special mount on a 3-axis linear translation stage (Newport M-565-XYZ). For obtaining a volume scan the sample was translated through the light sheet at 45° angle (along *t*-axis) using a PI high-resolution linear actuator (M-230.10). Images were recorded with a Hamamatsu ORCA-Flash4.0 V2 sCMOS camera. The microscope was controlled using the Micro-Manager software package^27^. Further, the acquired images were analysed and processed in Fiji^28^ and using custom scripts written in Python.

### Airy phase mask

The refractive phase mask with a continuous cubic surface profile was designed to generate the 1D Airy beams. The phase mask provides cubic modulation which is roughly proportional to the wavenumber and placed on the back focal plane of the excitation objective, this can be used to generate laterally nondispersing broadband Airy beams^22^. The mask is divided into four region which each can be used to generate an Airy beam with different parameter *α* = 3.5, 7, 14, and 21. The *α* parameter corresponds to the phase difference between the centre and edge of the aperture (*r* = 3.5 mm) in full wave cycles for *λ* = 532 nm. The phase plate was fabricated out of a fused silica substrate by PowerPhotonic LightForge service.

### Point spread function analysis

The two-photon fluorescence PSF and MTF of Gaussian and Airy light sheets were calculated from the corresponding pupil function as the scalar point spread function. The spectrum of the pulse was assumed to be Gaussian with a centre wavelength of 800 nm and a width corresponding to the duration of the simulated pulse. A Gaussian beam profile that had been focused to a line with a cylindrical lens was estimated on the pupil plane of the excitation objective. Both, the dispersion of the substrate material of the cubic phase mask, as well as of the immersion medium were taken into account in the simulations. Other than that, it is assumed that the dispersion of lenses and the excitation objective is perfectly compensated and a transform limited pulse is provided. In addition, sample-induced dispersion is not taken into account within this study. Due to difficulty of matching the exact experimental contitions with simulation paramters, the simulated PSF analysis is only used to provide a best- case scenario estimates for the achievable resolution.

For analysing the experimental PSF of the microscope the following procedure was used: a test sample of small, 0.2 or 0.4 μm, green or red fluorescing polystyrene microspheres was made by embedding them in 1.5% agarose. Image stacks were acquired using fine translations, Δ*t* = 92 nm, of the sample. The dataset was analysed using the Trackpy particle tracking toolkit^29^, written in Python, to retrieve accurate estimation of the angle between the actual translation axis and the optical axis of the detection objective. The respective shift was applied to the images in acquired stacks to transform them into the coordinate system of the detection objective. Thereafter, particle tracking data was used to extract the experimental PSF data at multiple locations and averaged over the subregions of the FOV. This experimental PSF was used to deconvolve the data acquired in the single-photon regime with Airy light sheet, while in the two-photon regime, deconvolution was not applied.

### Animals and tissue preparation

All experiments were approved by the UK Home Office and the University of St Andrews Animal Welfare Ethics Committee and conducted in accordance with the UK Animals (Scientific Procedure) Act, 1986. Food and water were provided ad libitum and there was 12 hour light/dark exposure.

Brain tissue from adult Sprague Dawley rats was fixed by transcardial perfusion with PBS followed by 4% paraformaldehyde (PFA) and postfixation in 4% PFA. Cryoprotection was performed overnight in 30% sucrose in 0.1 M PB. Brain tissue was sectioned sagitally on a sliding microtome (Leica) at a thickness of 40 μm. Sections were washed with PBS and blocked in 10% normal goat serum and 0.4% triton X-100 in PBS. Primary antibodies, anti-NCAM (neural cell adhesion molecule, 1:200, mouse monoclonal, Santa Cruz Biotechnology), and anti-GFAP (glial fibrillary acidic protein, 1:500, rabbit polyclonal, DAKO) were incubated in blocking buffer overnight at 4 °C, followed by incubation with secondary antibodies (1:500, goat anti-mouse or rabbit Alexa Fluor 568 or 488; Invitrogen), and mounting onto gelatin-coated slides.

### Data availability

The research data and materials supporting this publication can be accessed at: “Integrated single- and two-photon light sheet microscopy using accelerating beams (dataset)” (http://dx.doi.org/10.17630/1a525093-9612-4245-a0c3-42172709b97b).

## Electronic supplementary material

Supplementary Video 1

Supplementary Video 2

Supplementary Video 3

Supplementary Video 4

Supplementary Video 5

Supplementary info
