# Supplementary material for: Integrated single- and two-photon light sheet microscopy using accelerating beams
Source: Sci Rep. 2017 May 3;7:1435. doi: 10.1038/s41598-017-01543-4 (PMC5431168; doi:10.1038/s41598-017-01543-4)
Supplement: Supplementary file 6 — Supplementary info [file 41598_2017_1543_MOESM6_ESM.pdf]

Integrated single- and two-photon light sheet microscopy using accelerating beams: Supplementary Information

Peeter Piksarv[1,2,*], Dominik Marti[3], Tuan Le[4], Angelika Unterhuber[5], Lindsey H. Forbes[6], Melissa R. Andrews[6], Andreas Stingl[4], Wolfgang Drexler[5], Peter E. Andersen[3], and Kishan Dholakia[1,*]

[1]SUPA, School of Physics and Astronomy, University of St Andrews, North Haugh, St Andrews, KY16 9SS, UK
[2]Institute of Physics, University of Tartu, W. Ostwaldi 1, Tartu, 50411, Estonia
[3]Department of Photonics Engineering, Technical University of Denmark, Frederiksborgvej 399, 4000 Roskilde, Denmark
[4]Femtolasers Produktions GmbH, Fernkorngasse 10, 1100 Vienna, Austria
[5]Center for Medical Physics and Biomedical Engineering, Medical University Vienna, Waehringer Guertel 18-20, 1090 Vienna, Austria
[6]School of Medicine, University of St Andrews, North Haugh, St Andrews, KY16 9TF, UK
[*]kd1@st-andrews.ac.uk

February 27, 2017

Contents

Supplementary Note 1: Experimental comparison of the photobleaching rate 2

Supplementary Table S2: Imaging conditions for all the experiments 4

Supplementary Note 1: Experimental comparison of the photobleaching rate

The impact of the continued laser exposure to samples in the two-photon Airy light sheet microscope was measured on a sample of agarose-embedded green fluorescing microspheres with a diameter of 4.9 µm. The beads were imaged continuously over a time period of 25 minutes keeping the excitation shutter open over the duration. The sample was positioned in the focus of the light sheet and snapshot images were acquired with 5 second intervals. The mean drift of an particle in the image plane was ensured to be less than 1.1 µm. The Laser power was kept at the maximum level over the whole route of the measurement which meant that the total power at the sample plane over the cross-sectional area of the light sheet was 1.1 W. As the fluorescence signal level can depend on a particular particle the measurements were repeated for several different fluorescent beads. Figure S1 shows the experimental results for Airy and Gaussian light sheets in two-photon fluorescence regime. An exponential model $I(t) = a\exp(-bx) + c$ was fitted to the obtained fluorescence intensity curves to extract fluorescence bleaching rate and half-life values for each light sheet type. The experimental results are summarized in Table S1. From the results it can be seen that the mean photo-bleaching rate is 35% lower for the Airy light sheet compared to the Gaussian light sheet. However, these match within confidence intervals.

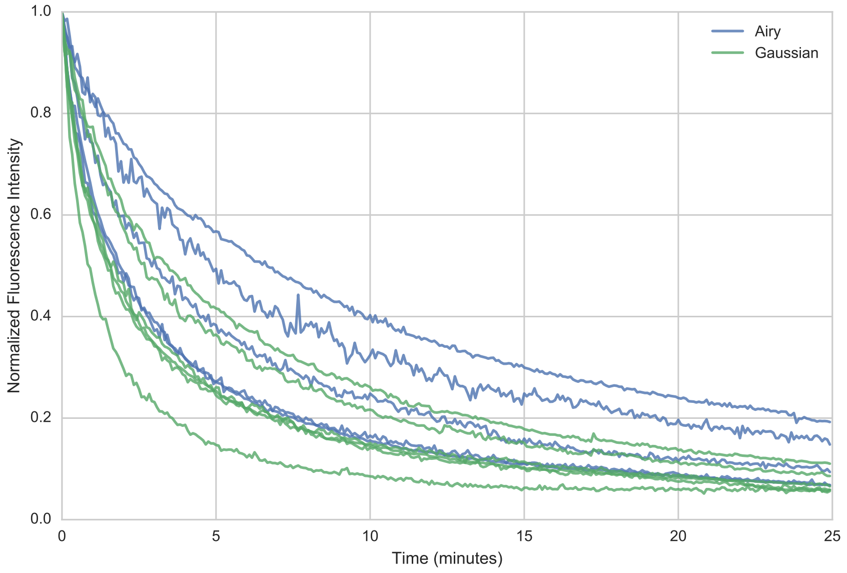

Supplementary Figure S1: Observed normalized two-photon fluorescence signal intensity over time for static Airy and Gaussian light sheets.

Light Sheet Type	Bleaching Rate (min^{-1})	Half Life (min)
Airy	0.22 ± 0.09	3.6 ± 1.7
Gaussian	0.34 ± 0.17	2.4 ± 1.1

Supplementary Table S1: Comparison of the photobleaching rates and fluorescence signal half-life for static two-photon Airy and Gaussian light sheet types. The uncertainties are given at 95% confidence level.

Supplementary Table S2: Imaging conditions for all the experiments

	Fig 3	Fig 4b	Fig 4b	Fig 5a	Fig 5b	Fig 5c	Fig 6a	Fig 6b
Laser	Core 2	HighQ-2	HighQ-2	HighQ-2	HighQ-2	Sapphire	Sprite XT	Sprite XT
Excitation wavelength (nm)	780	1,045	1,045	1,045	1,045	488	770	770
Power (mW)	93	917	917	917	917	1	780	780
Pulse duration (fs)	20	250	250	250	250	CW	150	150
Light-sheet mode	Various	Gaussian	Airy ($\alpha = 7$)	Gaussian	Airy ($\alpha = 7$)	Airy ($\alpha = 7$)	Gaussian	Gaussian
Exposure time (ms)	500	500	2,000	500	500	50	300	300
Filters	700/SP, 529/28	700/SP	700/SP	700/SP	700/SP	488 notch	650/SP, 450/40	650/SP, 529/28
Step size (µm)	0.1	0.46	0.46	0.46	0.46	0.46	0.46	0.46

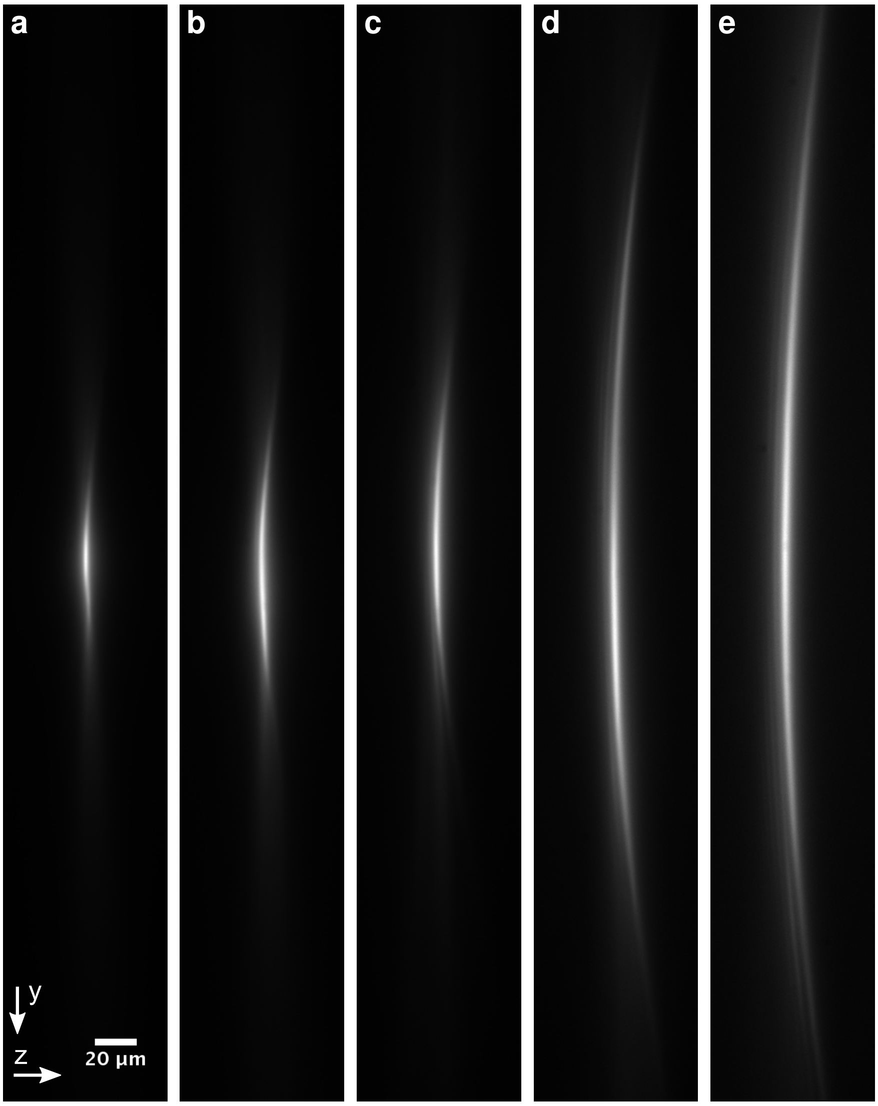

Supplementary Figure S2: Two-photon fluorescence light sheet profiles imaged from the side in a fluorescein solution in water. (a) Gaussian light sheet, (b)-(e) Airy light sheet with increasing α parameter (3.5, 7, 14, and 21) for cubic phase modulation depth. Imaged using a Coherent Mira fs-oscillator.
